# Impact of anti-TNF and anti-IL-12/IL-23 antibody therapy on periodontal inflammation and gingival crevicular fluid IL-6 in patients with inflammatory bowel diseases

**DOI:** 10.3389/fimmu.2026.1832952

**Published:** 2026-06-29

**Authors:** Nicole Neurath, Andre Jefremow, Selina Sitte, Hady Haririan, Raja Atreya, Marco Kesting

**Affiliations:** 1 Department of Oral and Cranio-Maxillofacial Surgery, Uniklinikum Erlangen, Friedrich-Alexander University Erlangen-Nürnberg, Erlangen, Germany; 2 Department of Medicine 1, Uniklinikum Erlangen, Friedrich-Alexander University Erlangen-Nürnberg, Erlangen, Germany; 3 Deutsches Zentrum Immuntherapie DZI, Uniklinikum Erlangen, Erlangen, Germany; 4 Department of Periodontology, Dental Clinic, Faculty of Medicine, Sigmund Freud University, Vienna, Austria

**Keywords:** cytokine targeting, cytokines, oral mucosa, pathogenesis, periodontitis

## Abstract

**Introduction:**

Periodontitis prevalence is elevated in patients with inflammatory bowel diseases (IBD). Biological therapies targeting cytokines such as tumor necrosis factor (TNF) and interleukin-12/23 (IL-12/23) are central to IBD management, yet their impact on periodontal health remains unclear.

**Methods:**

In a cross-sectional, hypothesis-generating design, 45 patients with IBD were examined and stratified into three groups according to their biological therapy: anti-TNF therapy (n = 15), anti-IL-12/23 therapy (n = 15), and a control group not receiving these biologics (n = 15). Periodontal status was assessed using the Periodontal Screening Index (PSI; codes 0–4) across all sextants as a standardized screening measure. Medical history, oral hygiene behavior, and prior periodontal diagnoses were documented. To assess local immune activity, interleukin-6 (IL-6) concentrations in gingival crevicular fluid (GCF) were quantified by enzyme-linked immunosorbent assay (ELISA).

**Results:**

Overall, 16 patients exhibited either known or previously undiagnosed periodontitis, with interindividual variability in severity. Patients receiving anti-IL-12/23 therapy exhibited lower mean and maximal PSI scores compared with anti-TNF–treated patients, and no sextants with advanced periodontal inflammation (PSI 3–4) were detected in this group. In contrast, active or latent periodontitis was observed in both the anti-TNF and control cohorts. Concordantly, IL-6 levels in GCF were significantly reduced in patients undergoing IL-12/23 blockade compared with controls, indicating attenuated local inflammatory signaling at the periodontal interface. These findings were observed despite no statistically significant differences in clinical or endoscopic disease activity between groups.

**Conclusions:**

These exploratory findings suggest that systemic inhibition of the IL-12/23 axis in IBD is associated with reduced periodontal inflammatory burden and decreased local IL-6 activity, supporting a role for IL-23–dependent immune pathways in linking intestinal and oral mucosal inflammation. While causality cannot be inferred from this cross-sectional study, the data provide a mechanistic rationale for further longitudinal investigations into the impact of cytokine-targeted biologic therapy on the oral–gut immune axis.

## Introduction

Patients with inflammatory bowel diseases (IBD), including Crohn’s disease and ulcerative colitis, exhibit a pathophysiological dysregulation of mucosal immunity driven by genetic and environmental factors (1, 2). This immune imbalance results in excessive cytokine production, contributing to disease complications such as rectal bleeding, strictures or colorectal cancer (3, 4). Notably, patients with IBD show a higher prevalence of periodontitis than the general population, suggesting shared immunopathological mechanisms in the oral and intestinal mucosa (5–9). This raises the question whether therapeutic modulation of cytokines in IBD could influence periodontal disease activity.

Periodontitis, a leading cause of tooth loss worldwide, is characterized by local inflammation of the periodontium and progressive destruction of alveolar bone driven by a dysregulated host immune response to polymicrobial infection (10, 11). Historically classified as chronic or aggressive, periodontitis is now unified under a single category with multidimensional staging and grading based on clinical attachment loss, probing depth, radiographic bone loss, and disease progression (12, 13). The periodontium, composed of collagen-rich connective tissue anchoring teeth to alveolar bone, is essential for oral function. Disease develops stepwise, from mild gingival inflammation and pocket formation to advanced stages with abscesses, extensive bone loss, and tooth mobility. Modifiers such as smoking, diabetes, and poor oral hygiene can accelerate disease progression (14–16).

Cytokines, small regulatory proteins produced by immune and non-immune cells, are central to periodontitis pathogenesis (17, 18). In healthy gingiva, a balance between pro- and anti-inflammatory cytokines maintains tissue integrity. Periodontitis disrupts this balance, with elevated IL-1β, TNF, IL-6, IL-17, and IFN-γ promoting local inflammation, tissue degradation, and bone resorption (18–21). Cytokines mediate alveolar bone loss via upregulation of RANKL and suppression of osteoprotegerin, activating osteoclasts. They also stimulate matrix metalloproteinases and chemokines in fibroblasts, epithelial cells, and immune cells, amplifying extracellular matrix degradation and leukocyte recruitment.

Among the cytokines driving periodontitis, tumor necrosis factor (TNF) is a pivotal pro-inflammatory mediator produced by macrophages, lymphocytes, and other immune cells (22–24). TNF signals through TNFR1 and TNFR2, regulating immune cell recruitment, activation, survival, tissue injury, and angiogenesis. Elevated TNF levels are observed locally in periodontal lesions and systemically in periodontitis patients, correlating with bacterial load and disease severity. Experimental models demonstrate that TNF-TNFR1 signaling drives neutrophil recruitment, RANKL expression, and osteoclast activation, while TNF blockade reduces inflammation, tissue injury, and alveolar bone loss (18, 25), highlighting TNF’s central role in local immune regulation.

Interleukin-23 (IL-23), a heterodimeric cytokine of the IL-12 family, is produced by antigen-presenting and epithelial cells in response to microbial stimuli (26). IL-23 promotes Th17 differentiation and IL-17 production, amplifying local inflammation. In periodontitis, IL-23 and IL-17 levels are elevated in gingival crevicular fluid (GCF) and correlate with disease severity (20, 27, 28). Both hematopoietic and non-hematopoietic cells, including epithelial and periodontal ligament fibroblasts, contribute to IL-23 production, regulated by microbial Toll-like receptor signals and pro-inflammatory cytokines such as IL-1β. Functionally, IL-23 enhances RANKL expression in osteoblasts, promoting osteoclast-mediated bone resorption (27, 29). Periodontitis also contributes to systemic inflammation. Elevated circulating IL-1β, TNF, and IL-6 levels link periodontal disease to comorbidities such as arthritis, neurodegeneration, and IBD (18, 24, 30). Conversely, systemic inflammatory conditions, such as rheumatoid arthritis and IBD, increase susceptibility to periodontitis, indicating bidirectional crosstalk between oral immunity and systemic inflammatory pathways.

Given the higher prevalence of periodontitis in IBD and the critical roles of TNF and IL-23 in both intestinal and periodontal inflammation, cytokine-targeted therapies in IBD may modulate periodontal disease. Biologics that neutralize TNF or IL-12/23, including infliximab, adalimumab, ustekinumab, mirikizumab, and risankizumab, are central in IBD management (31, 32). This suggests the hypothesis that systemic blockade of TNF or IL-12/23 could reduce concurrent periodontitis severity. Specifically, it remains to be explored whether anti-TNF or anti-IL-12/23 therapies decrease periodontal pocket depth compared to patients not receiving cytokine-blocking agents, and whether IL-12/23 inhibition exerts superior effects relative to TNF blockade. Understanding these mechanisms may provide critical insight into the crosstalk between systemic cytokine modulation and oral mucosal immunity in IBD patients.

## Methods

### Study design and ethics approval

This study was conducted as an exploratory, hypothesis-generating clinical observational investigation to evaluate the association between biological therapy and the inflammatory burden of periodontitis in patients with IBD. This was a prospective, cross-sectional non-interventional study. Because no prior data were available on this specific research question, no formal sample size calculation was feasible. The study received ethical approval from the Friedrich-Alexander University Erlangen-Nürnberg Ethics Committee (approval number 24-431B) on November 22, 2024. Written informed consent was obtained from all participants before enrollment.

### Study objectives and hypotheses

The primary objective was to determine whether anti-cytokine therapy in IBD influences the prevalence and severity of periodontitis. Secondary objectives included evaluating whether anti-IL-12/23 therapy exerts stronger effects than anti-TNF therapy or non-biologic treatments, and whether biologic therapy modifies cytokine concentrations in GCF.

### Participants

Participants were recruited from the IBD specialty outpatient clinic at Universitätsklinikum Erlangen between December 1, 2024, and November 1, 2025. Inclusion criteria comprised adults ≥18 years with a confirmed diagnosis of IBD (Crohn’s disease or ulcerative colitis) and the ability to provide informed consent. Exclusion criteria included malignancies, other autoimmune disorders (e.g., systemic lupus erythematosus), recent antibiotic therapy, chemotherapy, head and neck radiation, antiresorptive therapy, and conditions requiring preoperative antibiotic prophylaxis (e.g., prosthetic heart valves, history of endocarditis). These criteria were designed to ensure patient safety and study integrity.

### Study groups and sample size rationale

Participants were assigned to one of three groups according to their IBD therapy: (i) anti-IL-12/23 therapy (e.g., ustekinumab and biosimilars, guselkumab, mirikizumab, risankizumab), (ii) anti-TNF therapy (e.g., infliximab and biosimilars, adalimumab and biosimilars, golimumab), or (iii) controls without anti-TNF and anti-IL-12/23 biologic therapy. Each group included 15 patients, a pragmatic choice for this exploratory study. While this sample size was not statistically powered to detect small effects, it was considered sufficient to identify trends and potential group differences, while remaining feasible within the recruitment timeframe.

### Clinical and periodontal assessment

All participants underwent a comprehensive dental examination, including evaluation of the periodontal status using the Periodontal Screening Index (PSI; codes 0–4). Tooth loss, probing depth, clinical attachment level, and gingival health were assessed. Demographic and medical history data were collected, including IBD type, disease duration, current and prior therapies, and risk factors for periodontitis such as smoking, diabetes, stress, and oral hygiene practices. Patients with a previous diagnosis of periodontitis had undergone prior subgingival debridement therapy. Clinical and endoscopic activity of IBD was determined by established clinical activity scores (33–35).

Endoscopic disease activity was categorized according to established scoring systems (33, 36). For ulcerative colitis, endoscopic activity was classified using the Mayo endoscopic subscore (0 = no activity, 1–2 = mild/moderate activity, 3 = high activity). For Crohn’s disease, SES-CD scores were categorized as 0–2 (no activity), 3–15 (mild/moderate activity), and >15 (high activity).

### Gingival crevicular fluid collection

GCF samples were obtained using sterile paper strips placed for 30 seconds into the periodontal sulcus at two sites: the mesial aspect of the first maxillary molar and the distal aspect of the mandibular canine. These standardized anatomical sites were selected to ensure reproducibility and comparability across patients. Within these predefined regions, GCF samples used for IL-6 analysis were analyzed from the site exhibiting the highest PSI score. This minimally invasive procedure was performed under standardized conditions, and samples were stored for subsequent cytokine analyses.

### General oral hygiene assessment

Participants’ oral hygiene practices were evaluated using a structured questionnaire. Data included toothbrushing frequency, use of interdental brushes or floss, mouthwash use, and the interval since the last professional dental cleaning. These measures were considered to account for potential confounding effects on periodontal status and local cytokine concentrations.

### IL-6 measurement in GCF

IL-6 concentrations were measured using an ELISA kit (ThermoFisher Scientific, Cat. No. BMS213-2HS) according to the manufacturer’s protocol. Samples and standards were added in duplicate to pre-coated wells, incubated, and washed. A biotinylated detection antibody and streptavidin-HRP were applied sequentially, and the reaction was developed with TMB substrate. Optical density was measured at 450 nm, and IL-6 concentrations were determined from a standard curve. The assay sensitivity was 0.08 pg/mL.

### Data management

All patient data were pseudonymized to ensure confidentiality. No identifiable data were shared with third parties. Participants were informed they could withdraw at any time without consequence. GCF samples were securely stored at the IBD clinic for future analyses and were not distributed externally.

### Statistical analysis

Data were analyzed using non-parametric tests due to non-normal distributions. A Kruskal–Wallis test was applied to compare values across the three groups (control, TNF, IL-12/23). Dunn’s *post-hoc* test with Bonferroni correction was used for pairwise comparisons, with reported p-values interpreted exploratorily. Descriptive statistics summarized demographic, clinical, and laboratory data. Graphical analyses were performed using GraphPad Prism and Miro software.

## Results

### Immunological and clinical characterization of the IBD cohort

A total of 45 patients with clinically diagnosed inflammatory bowel disease (IBD) treated at the specialized IBD outpatient clinic of the University Hospital Erlangen were included in this study (**Table 1**). The cohort consisted of 26 patients with Crohn’s disease (CD), 18 patients with ulcerative colitis (UC), and one patient with indeterminate colitis. 23 participants were male and 22 female. The mean age at study inclusion was 46.6 ± 14.5 years (range 24–77 years), and mean disease duration was 16.9 ± 12.0 years, reflecting a predominance of long-standing immune-mediated disease in the intestine.

**Table 1:** Patient characteristics including study group, age, diagnosis, disease localization, disease duration, medication, and the presence or absence of known periodontitis. Kruskal–Wallis tests revealed no significant differences between the three groups with respect to age (mean ± SEM: control group 54.1 ± 3.7 years; anti-TNF group 43.8 ± 4.2 years; anti-IL-12/23 group 41.3 ± 3.3 years) or disease duration (mean ± SEM: control group 17.7 ± 2.7 years; anti-TNF group 17.3 ± 3.1 years; anti-IL-12/23 group 15.7 ± 3.4 years).

No.	Group	Age	Diagnosis	Inflammation	Duration	Medication	Endoscopic activity	Known
1	Control group	71	Ulcerative colitis	Colitis	9 years	Vedolizumab	No	No
2	Control group	48	Crohn's disease	Ileitis, Colitis	10 years	No	No	No
3	Control group	29	Ulcerative colitis	Colitis	6 years	Upadacitinib	High	No
4	Control group	39	Crohn's disease	Colitis	15 years	Vedolizumab	High	No
5	Control group	49	Crohn's disease	Ileitis, Colitis	18 years	Upadacitinib	High	No
6	Control group	35	Ulcerative colitis	Colitis	13 years	M/M	No	No
7	Control group	87	Ulcerative colitis	Colitis	N/A	Vedolizumab	High	No
8	Control group	45	Indeterminate	Colitis	24 years	Upadacitinib	M/M	No
9	Control group	56	Crohn's disease	Ileitis, Colitis	28 years	Upadacitinib	No	No
10	Control group	73	Crohn's disease	Colitis	29 years	Vedolizumab	No	Yes
11	Control group	43	Ulcerative colitis	Colitis	16 years	Prednisolone	No	Yes
12	Control group	68	Ulcerative colitis	Colitis	28 years	Vedolizumab	No	Yes
13	Control group	63	Crohn's disease	Ileitis	18 years	Vedolizumab	No	Yes
14	Control group	77	Ulcerative colitis	Colitis	35 years	M/M	N/A	No
15	Control group	55	Crohn's disease	Colitis	1 year	Prednisone	M/M	Yes
16	Anti-TNF group	33	Crohn's disease	Ileitis	14 years	Infliximab	N/A	No
17	Anti-TNF group	55	Crohn's disease	Colitis	34 years	Infliximab	N/A	Yes
18	Anti-TNF group	38	Crohn's disease	Ileitis, Colitis	14 years	Infliximab	No	No
19	Anti-TNF group	33	Ulcerative colitis	Colitis	15 years	Infliximab	M/M	No
20	Anti-TNF group	40	Crohn's disease	Ileitis, Colitis	15 years	Adalimumab	M/M	No
21	Anti-TNF group	35	Crohn's disease	Colitis	10 years	Infliximab	M/M	No
22	Anti-TNF group	27	Crohn's disease	Colitis	9 years	Infliximab	M/M	No
23	Anti-TNF group	26	Crohn's disease	Colitis	3 years	Infliximab	No	No
24	Anti-TNF group	49	Crohn's disease	Colitis	30 years	Infliximab	No	No
25	Anti-TNF group	72	Ulcerative colitis	Colitis	21 years	Infliximab	M/M	No
26	Anti-TNF group	24	Crohn's disease	Ileitis	3 years	Infliximab	M/M	No
27	Anti-TNF group	39	Crohn's disease	Ileitis, Colitis	17 years	Adalimumab	No	No
28	Anti-TNF group	68	Ulcerative colitis	Colitis	50 years	Adalimumab	No	Yes
29	Anti-TNF group	48	Ulcerative colitis	Colitis	9 years	Adalimumab	No	No
30	Anti-TNF group	68	Crohn's disease	Ileitis	16 years	Adalimumab	M/M	Yes
31	Anti-IL-12/23 group	42	Ulcerative colitis	Colitis	3 years	Ustekinumab	M/M	No
32	Anti-IL-12/23 group	32	Ulcerative colitis	Colitis	6 years	Risankizumab	High	Yes
33	Anti-IL-12/23 group	30	Crohn's disease	Colitis	2 years	Risankizumab	M/M	No
34	Anti-IL-12/23 group	26	Crohn's disease	Ileitis, Colitis	13 years	Ustekinumab	No	No
35	Anti-IL-12/23 group	70	Crohn's disease	Ileitis, Colitis	54 years	Risankizumab	M/M	Yes
36	Anti-IL-12/23 group	43	Crohn's disease	Ileitis	13 years	Ustekinumab	M/M	No
37	Anti-IL-12/23 group	31	Crohn's disease	Ileitis	9 years	Risankizumab	No	No
38	Anti-IL-12/23 group	43	Crohn's disease	Ileitis	13 years	Ustekinumab	No	No
39	Anti-IL-12/23 group	35	Crohn's disease	Ileitis	7 years	Ustekinumab	M/M	No
40	Anti-IL-12/23 group	46	Crohn's disease	Ileitis, Colitis	26 years	Risankizumab	No	No
41	Anti-IL-12/23 group	61	Crohn's disease	Ileitis	40 years	Ustekinumab	M/M	No
42	Anti-IL-12/23 group	30	Crohn's disease	Colitis	12 years	Ustekinumab	No	No
43	Anti-IL-12/23 group	33	Ulcerative colitis	Colitis	6 years	Ustekinumab	M/M	Yes
44	Anti-IL-12/23 group	59	Crohn's disease	Ileitis, Colitis	26 years	Risankizumab	M/M	No
45	Anti-IL-12/23 group	43	Crohn's disease	Colitis	5 years	Risankizumab	M/M	No

Patients were stratified according to immunomodulatory biological therapy. Fifteen patients received tumor necrosis factor (TNF) inhibitors (adalimumab or infliximab; Anti-TNF group), 15 patients were treated with IL-12/IL-23 pathway inhibitors (ustekinumab or risankizumab; Anti-IL-12/IL-23 group), and 15 patients served as controls without TNF or IL-12/IL-23 blockade. Therapies in the control group included corticosteroids, vedolizumab, the JAK inhibitor upadacitinib, or no immunomodulatory treatment (**Table 1**). This design allowed comparison of distinct cytokine-targeted immune interventions.

**Figure 1:**
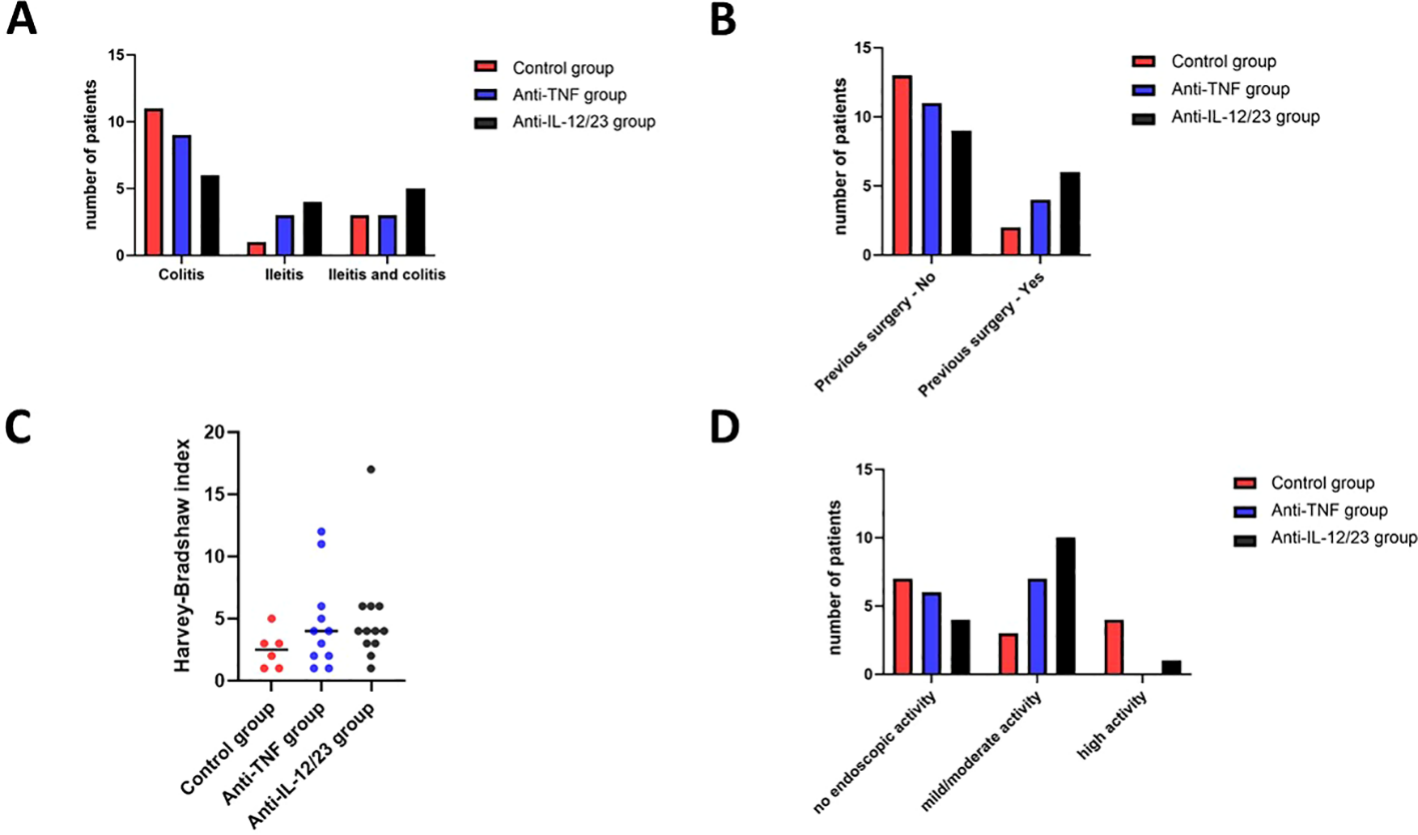
Clinical characterization of IBD patients. Clinical characterization of the patients included in the study. The clinical IBD manifestation (ileitis, colitis, or both) in the three groups (control, anti-TNF, anti-IL-12/IL-23) is shown [panel **(A)**]. In addition, information on previous IBD-related surgeries (e.g., ileocecal resection) is presented [panel **(B)**]. Finally, data on the Harvey–Bradshaw Index [HBI, panel **(C)**] as well as on endoscopic inflammatory activity of IBD [panel **(D)**; number of patients with endoscopic disease activity] in the three groups are detailed.

Colonic involvement was the predominant disease manifestation across all groups, consistent with sustained immune cell activation at the colonic mucosa (**Figure 1A**). Combined ileocolonic disease and isolated ileal involvement were less frequent. A relevant proportion of patients in all groups had previously undergone intestinal surgery, indicating a history of severe or refractory inflammation (**Figure 1B**). The number of previous surgeries was higher in the Anti-IL-12/IL-23 group as compared to the other groups. However, no significant difference in the clinical activity score (**Figure 1C**) and the endoscopic activity scores (**Figure 1D**) were noted between the three groups, suggesting comparable inflammatory activity. In fact, most patients had low Harvey-Bradshaw indices and absent to moderate endoscopic disease activity scores suggesting that clinical activity of IBD was well controlled in most cases. However, the distribution of endoscopic disease activity differed numerically between groups. Endoscopic remission was less frequent in the anti-IL-12/IL-23 group, whereas patients with high inflammatory activity were observed only in the control group. Notably, few cases of severe endoscopic inflammation were detected in either biologic treatment group. Sex distribution was comparable between groups.

### Periodontal inflammatory burden is reduced under IL-12/IL-23 blockade

To determine whether systemic cytokine-targeted therapy influences oral inflammatory status, periodontal health was assessed using the Periodontal Screening Index (PSI). PSI scores (0–4) were recorded for all sextants in all patients, resulting in 270 total measurements.

**Figure 2:**
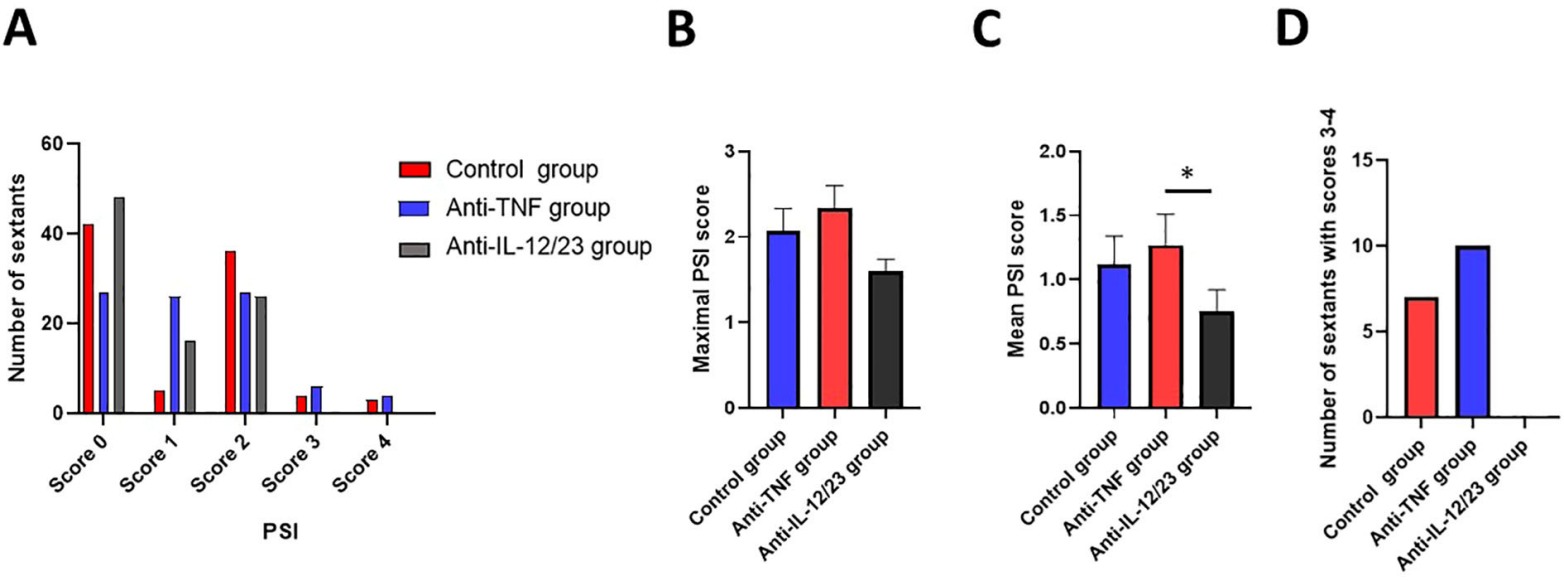
Periodontal screening index (PSI) in IBD patients. In all 45 IBD patients, the Periodontal Screening Index (PSI) was used to detect signs of periodontitis and to identify patients requiring further evaluation. The distribution of PSI scores across all sextants in the 45 patients is shown in panel **(A)**. In addition, the maximal PSI score per group +/- SEM (control, anti-TNF, anti-IL-12/IL-23) is shown [panel **(B)**]. Furthermore, the mean PSI scores +/- SEM in all three groups are detailed [panel **(C)**]. Finally, the number of sextants with PSI scores of 3–4 in the three groups (control, anti-TNF, anti-IL-12/IL-23) is shown [panel **(D)**], indicating active periodontitis. *, 0.05.

Mean PSI values per sextant differed between groups (**Figure 2A**). Patients receiving IL-12/IL-23 inhibitors exhibited the lowest periodontal inflammatory burden (mean ± SD: 0.75 ± 0.17), whereas higher values were observed in the control group (1.12 ± 0.22) and the Anti-TNF group (1.27 ± 0.24). Maximal and mean PSI scores were lower in the Anti-IL-12/IL-23 group as compared to the other groups (**Figures 2B, C**). In contrast to the control and the anti-TNF groups, no sextants with advanced periodontal inflammation (PSI scores 3 or 4) were detected in the Anti-IL-12/IL-23 group (**Figure 2D**).

A non-parametric Kruskal–Wallis test followed by exploratory Dunn’s *post-hoc* analysis showed that PSI values tended to be lower in the Anti-IL-12/IL-23 group compared with the Anti-TNF group (p = 0.055), whereas no differences were detected between Anti-TNF and control patients (p = 0.61) or between Anti-IL-12/IL-23 and control patients (p = 0.20). Maximal PSI values per patient ± standard error of the mean (SEM) were as follows: 2.07 ± 0.26 in the control group, 2.33 ± 0.27 in the Anti-TNF group, and 1.60 ± 0.14 in the Anti-IL-12/IL-23 group. These results support a trend toward reduced periodontal inflammation under IL-12/IL-23 blockade. Consistently, none of the patients in the Anti-IL-12/IL-23 group showed marked periodontal inflammation with scores 3-4, while several patients in the other groups showed scores 3-4 (**Figure 2D**).

### Inactive pre-existing periodontitis and no new cases of subclinical periodontitis in anti-IL-12/IL-23-treated IBD patients

A history of previously diagnosed periodontitis was reported in all groups (control group n= 6; anti-TNF group n= 3; anti-IL-12/IL-23 group n= 3). Newly identified, previously undiagnosed periodontitis (PSI ≥ 3) was detected in three control patients (20%) and two Anti-TNF patients (13.3%), but in none of the Anti-IL-12/IL-23–treated patients (0%). Importantly, two patients receiving IL-12/IL-23 inhibitors with a history of periodontitis exhibited PSI scores of 0 across all sextants at the time of examination, indicating effective suppression of active periodontal inflammation.

### Oral hygiene and risk factors for periodontitis

**Figure 3:**
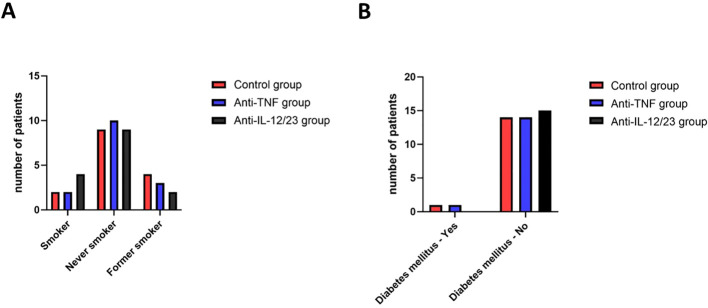
Risk factors for periodontitis in IBD patients. The presence or absence of known risk factors for periodontitis was assessed in 45 IBD patients (control group, anti-TNF group, anti-IL-12/IL-23 group). Specifically, patients were asked about smoking and diabetes mellitus. The presence or absence of smoking [panel **(A)**] and diabetes mellitus [panel **(B)**] in all three groups is indicated.

**Figure 4:**
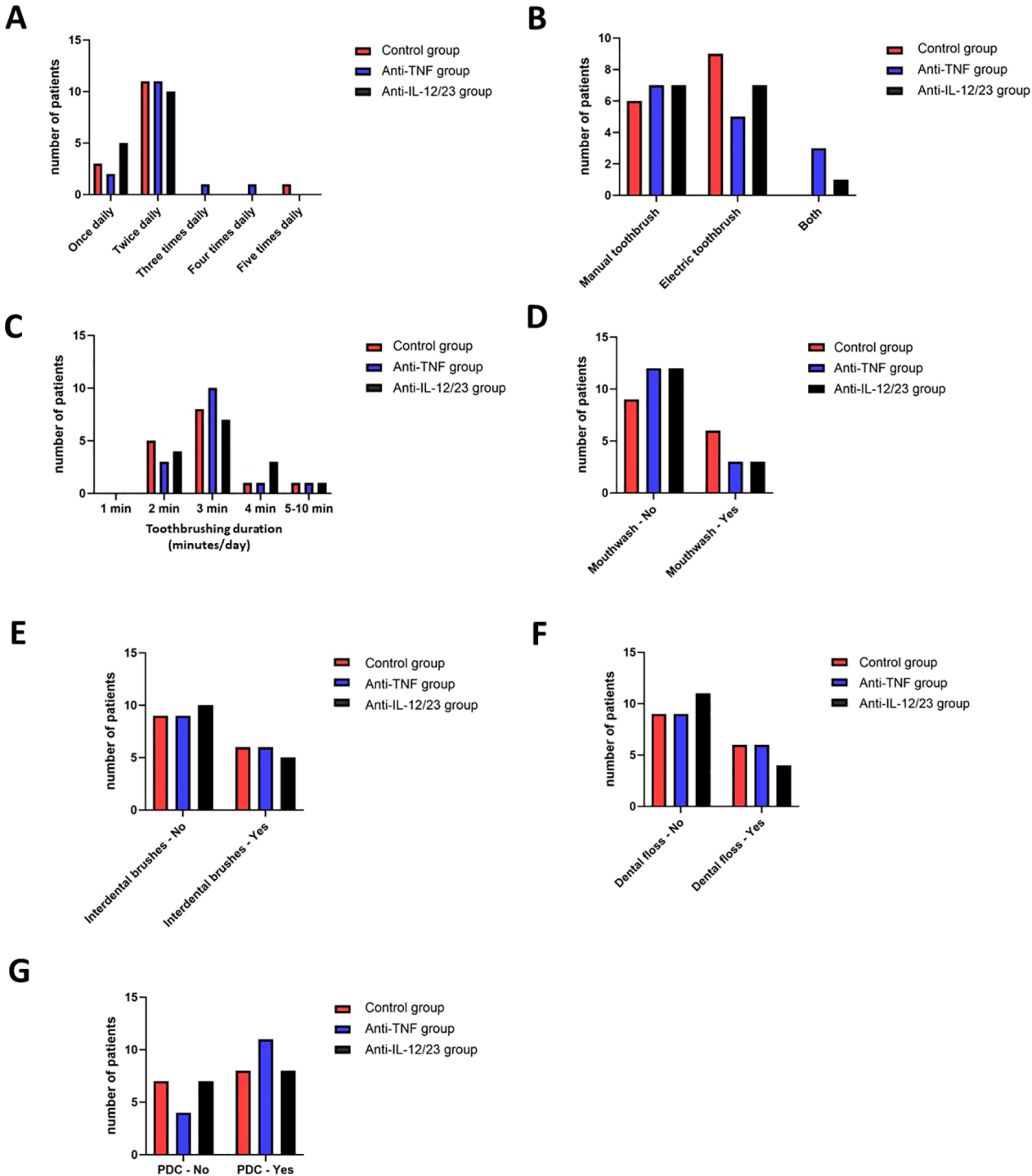
Oral hygiene habits in IBD patients. Oral hygiene habits in IBD patients across the three study groups (control, anti-TNF, anti-IL-12/IL-23) are shown. The daily toothbrushing frequency is presented in panel **(A)**, and the use of electric and manual toothbrushes is shown in panel **(B)** The average duration of toothbrushing in minutes per day is shown in panel **(C)** Information on the use of mouthwash [panel **(D)**], interdental brushes [panel **(E)**], and dental floss [panel **(F)**] is also presented. Finally, the use of professional dental cleaning (PDC) in the three groups is shown in panel **(G)**.

Classical risk factors for periodontitis, including smoking and diabetes mellitus type 2, were infrequent and similarly distributed across all groups (**Figures 3A, B**). Oral hygiene behaviors, such as frequency and duration of tooth brushing, use of dental floss, interdental brushes, mouth rinses, and professional dental cleaning, were largely comparable between groups (**Figures 4A–G**). Notably, Anti-TNF–treated patients reported the highest utilization of professional periodontal prophylaxis despite exhibiting higher mean PSI values. These findings suggest that lower levels of periodontal inflammation in the Anti-IL-12/IL-23 group were not primarily driven by hygiene behavior or metabolic risk factors.

### IL-6 levels in gingival crevicular fluid are reduced under IL-12/IL-23 blockade

**Figure 5:**
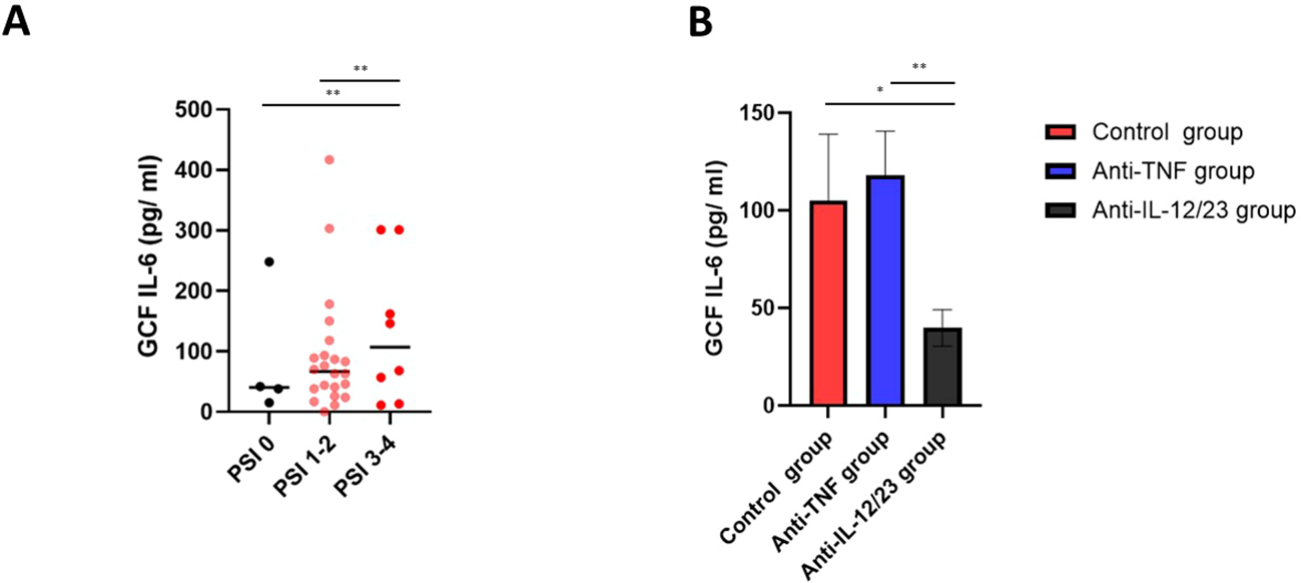
IL-6 levels in gingival GCF from IBD patients. GCF was collected from IBD patients and assessed for cytokine content by specific ELISA. IL-6 cytokine concentrations in IBD patients are shown in relation to PSI scores, indicating elevated IL-6 levels in patients with PSI scores of 3–4 compared to patients with scores of 0–2 (*p < 0.05; **p < 0.01) [panel **(A)**]. Moreover, IL-6 levels in GCF were determined in all three groups [mean values +/- SEM in control, anti-TNF, anti-IL-12/IL-23 groups; panel **(B)**], demonstrating significantly reduced levels in anti-IL-12/23-treated patients compared to anti-TNF-treated patients and control patients (*p < 0.05; **p < 0.01).

To further characterize local immune activation at the oral mucosal interface, interleukin-6 (IL-6) concentrations were measured in GCF using ELISA. IL-6 was detectable in GCF samples in most patients across all treatment groups. IL-6 levels were higher in patients with PSI scores of 3–4 as compared to patients with PSI scores of 0 or 1-2 (**Figure 5A**). A Kruskal–Wallis test revealed a significant difference among these groups (H(2) = 18.7, p < 0.001). *Post-hoc* Dunn tests indicated that PSI group 3–4 had significantly higher values compared to both PSI group 0 (p = 0.004) and PSI group 1-2 (p = 0.001), whereas there was no significant difference between PSI groups 0 and 1-2 (p = 0.62). These findings suggested that higher PSI scores of 3–4 are associated with increased GCF IL-6 levels. These results support a concordant relationship between clinical periodontal inflammation and local mucosal immune activation.

In addition, IL-6 concentrations in GCF were significantly higher in the control group and in the Anti-TNF group compared with patients receiving IL-12/IL-23 inhibitors (**Figure 5B**). In contrast, no significant difference in IL-6 levels was observed between the anti-TNF group and the control group. A non-parametric Kruskal–Wallis test demonstrated a statistically significant difference among the three groups, *H*(2) = 8.72, *p* = .013. For *post hoc* analysis, pairwise Dunn tests were performed with Bonferroni correction to adjust for multiple comparisons (adjusted α = .0167). These analyses revealed statistically significant differences between the anti-IL-12/IL-23 group and both the control group (*p* = .014) and the anti-TNF group (*p* = .006). In contrast, no statistically significant difference was observed between the control group and the anti-TNF group (*p* = .29). These findings suggest that selective inhibition of the IL-12/IL-23 axis is associated with reduced local IL-6 levels, whereas TNF blockade does not substantially modulate IL-6 concentrations in the gingival microenvironment as compared to the control group.

In summary, while systemic intestinal disease activity was largely controlled across all treatment groups, periodontal inflammatory burden and local IL-6 levels in GCF differed according to the targeted cytokine pathway. Inhibition of the IL-12/IL-23 axis was consistently associated with reduced periodontal inflammation and lower IL-6 concentrations at the oral mucosal interface. In contrast, TNF blockade did not differ from control treatment with respect to periodontal status or local IL-6 levels. These findings support the concept of shared immunopathogenic mechanisms between intestinal and oral mucosal tissues and implicate IL-12/IL-23–dependent immune pathways in linking IBD with periodontal inflammation.

## Discussion

In this prospective, exploratory study, we investigated whether cytokine-targeted biologic therapies used for IBD are associated with differences in the prevalence and inflammatory activity of periodontitis. A total of 45 patients with Crohn’s disease or ulcerative colitis were clinically examined, including assessment of periodontal pocket depth using the Periodontal Screening Index (PSI), comprehensive oral hygiene anamnesis, and analysis of local cytokine levels in GCF. Patients were stratified into three groups according to their systemic immunomodulatory therapy: Anti-TNF therapy, Anti-IL-12/IL-23 therapy, and a control group without TNF or IL-12/IL-23 blockade. Twelve patients had a history of diagnosed periodontitis, and five additional patients showed previously undetected signs of periodontitis based on PSI assessment. Notably, newly identified periodontitis occurred exclusively in the control and anti-TNF groups, but not in patients receiving IL-12/IL-23 blockade, despite no evidence of lower, and partially even higher, endoscopic disease activity in this group, arguing against disease activity as the primary driver of this effect.

Both sextant-based and individual-level analyses revealed lower mean PSI values in the anti-IL-12/IL-23 group compared with the anti-TNF and control groups. Furthermore, no cases with PSI scores of 3–4 were detected in the former group as compared to the latter groups. Although the limited sample size precluded definitive conclusions, these findings are consistent with the concept that IL-12/IL-23 inhibition may suppress local periodontal inflammatory activity. In agreement with this concept, IL-6 concentrations in GCF were significantly reduced in patients receiving anti-IL-12/IL-23 therapy compared with both control and anti-TNF–treated patients, further supporting therapy-specific modulation of local immune responses. Taken together, the data suggest a potential anti-inflammatory effect of IL-12/IL-23 blockade on periodontal immune activation.

Both periodontitis and IBD are chronic inflammatory disorders driven by dysregulated host–microbe interactions at mucosal surfaces (37–40). Cytokine production by immune and stromal cells in both diseases is strongly induced by microbial antigens, and both the gut and the oral cavity represent highly colonized microbial ecosystems. Following the intestine, the oral cavity is the second most densely colonized microbial niche in humans, supporting the concept of a functional oral–gut axis (9, 41–43). Multiple studies have demonstrated that oral pathobionts can translocate to the intestine, contributing to gut dysbiosis and intestinal inflammation (44–46). Conversely, intestinal immune activation may influence oral tissues through circulating cytokines or immune cell trafficking (40, 43). Shared immunological features of periodontitis and IBD include neutrophil activation, T cell–mediated inflammation, and elevated local concentrations of pro-inflammatory cytokines such as TNF, IL-1β, IL-6, IL-17, and IL-23 (3, 20, 21, 47). Integrated transcriptomic analyses have identified overlapping inflammatory gene signatures in both diseases, highlighting a bidirectional immunopathogenic relationship (18, 43, 47). Particularly, the IL-23/Th17 axis has emerged as a central pathway linking mucosal inflammation at distinct anatomical sites. IL-23 promotes the differentiation, stabilization, and pathogenicity of Th17 cells, which produce IL-17A, IL-17F, and IL-6 and drive neutrophil recruitment, tissue destruction, and bone resorption (18, 20, 28, 47–52). These mechanisms are relevant for both intestinal and periodontal inflammation.

Biologic therapies targeting TNF and IL-12/IL-23 are well established in the treatment of IBD, yet their effects on extraintestinal inflammatory diseases, such as periodontitis, remain insufficiently characterized (53–57). In this study, anti-TNF therapy was not associated with reduced periodontal inflammatory burden. PSI values were comparable between anti-TNF–treated patients and controls, and advanced PSI scores were observed in both groups. This contrasts with studies in rheumatoid arthritis, where TNF inhibition has been associated with improvements in periodontal parameters (58, 59). Several explanations may account for this discrepancy. First, pathogenetic mechanisms may differ between rheumatoid arthritis and IBD-associated periodontitis. Second, the effect size of TNF blockade on periodontal inflammation may be modest and not detectable in a small cohort. Third, patients in the control group frequently received other anti-inflammatory therapies, potentially attenuating observable differences.

In contrast, IL-12/IL-23 blockade was associated with consistently lower PSI values and absence of advanced periodontal inflammation (scores 3-4). Importantly, two patients with a history of periodontitis in this group exhibited complete normalization of PSI scores. These findings are of particular interest, as IL-23 has been identified as a key cytokine in the pathogenesis of periodontitis, promoting Th17-driven inflammation, osteoclast activation, and alveolar bone loss (18, 27, 29, 51). IL-23 in periodontitis is produced by antigen-presenting cells and epithelial cells and elevated IL-23 levels in periodontal pockets have been shown to correlate with clinical attachment loss and disease severity in patients with periodontitis (60–63). Furthermore, the levels of the IL-23/IL-17 axis have been found to be positively correlated with the progression and severity of periodontal disease, as determined by probing depth, clinical attachment level, and gingival index (18). Therefore, blockade of IL-23 via specific neutralizing antibodies may ameliorate mucosal inflammation in periodontitis.

Analysis of IL-6 levels in GCF provided additional insight into the immunological effects of cytokine-targeted therapy. IL-6 is a pleiotropic cytokine produced by immune cells such as macrophages and T cells that amplifies Th17 responses, promotes neutrophil recruitment, and sustains chronic inflammation through classical and trans-signaling pathways (17, 64). Consistent with its pathogenic role in periodontitis, elevated IL-6 levels have been detected in GCF and periodontal lesions, where they correlate with disease severity, immune cell infiltration, and alveolar bone resorption in periodontitis (17, 65). Blockade of IL-6-dependent STAT3 activation *in vivo* (e.g. via tocilizumab) inhibited Th17 cytokine levels, periapical bone resorption and apical infiltration of immune cells such as macrophages highlighting the potential therapeutic relevance of targeting the IL-6/STAT3 signaling pathway (66, 67). Some studies also reported a decrease of IL-6 in the GCF after nonsurgical periodontal therapy suggesting that IL-6 may serve as a biomarker for response to therapy (68–70).

In the present study, IL-6 concentrations in GCF were significantly lower in patients receiving Anti-IL-12/IL-23 therapy compared with Anti-TNF–treated patients, whereas Anti-TNF therapy did not significantly affect local IL-6 levels as compared to control patients. This finding is immunologically relevant, as IL-23 is a key upstream regulator of pathogenic Th17 cells, which in turn promote local IL-6 production and IL-6–STAT3–dependent inflammatory circuits in periodontal tissues (66). Thus, IL-12/IL-23 blockade may indirectly attenuate IL-6–driven inflammation by limiting Th17 cell activation and maintenance.

The concordance between reduced IL-6 levels and lower PSI scores in the Anti-IL-12/IL-23 group supports the concept that systemic cytokine blockade can modulate local mucosal immune environments beyond the intestine. While IL-6 may also exert context-dependent regulatory functions, particularly in tissue homeostasis, the observed reduction of excessive IL-6 signaling likely reflects suppression of local inflammatory activity and provides a biologically plausible explanation for the reduced periodontal inflammatory burden under systemic IL-12/IL-23 blockade. Additional effects of IL-12/IL-23 neutralization on periodontitis in IBD may also arise from modulation of intestinal inflammation. In particular, oral bacteria such as the pathobiont *Porphyromonas gingivalis* can exacerbate oral pathobiont-induced periodontitis through generation of intestinal Th17 cells, which can migrate to and accumulate in the oral mucosa upon oral infection (oral–gut axis) (71).

This study has several limitations. The exploratory, hypothesis-generating design and limited sample size precluded formal power calculation and definitive statistical conclusions. The present study was not designed to provide detailed mechanistic insights into the immunological pathways linking periodontal and intestinal inflammation. In particular, no detailed functional analyses of IL-23/Th17-related pathways were performed. Therefore, the observed associations between cytokine-targeted therapy, PSI scores, and local IL-6 levels should be interpreted as exploratory and hypothesis-generating. Nevertheless, the concordant changes observed across clinical periodontal parameters and local inflammatory cytokine levels support the biological plausibility of a therapy-associated modulation of the oral-gut inflammatory axis. Patients were examined at a single time point, preventing longitudinal assessment of periodontal changes before and after therapy initiation. Comprehensive periodontal diagnostics, including radiographic assessment, were not feasible. Thus, future prospective studies with more detailed patient stratification based on periodontal staging and grading are warranted. Furthermore, patients in the control group received heterogeneous anti-inflammatory therapies, potentially reducing detectable differences. Finally, given the limited sample size and the exploratory nature of this study, formal multivariate adjustment for potential confounders (smoking, diabetes, duration of treatment) was not feasible. Despite these limitations, this study represents the first prospective clinical investigation addressing the impact of Anti-IL-12/IL-23 cytokine-targeted biologic therapy on periodontal inflammation in IBD. The parallel assessment of clinical periodontal parameters and local cytokine levels provides an integrated view of systemic and mucosal immune modulation.

In this exploratory study, anti-IL-12/IL-23 therapy in IBD was associated with reduced periodontal inflammatory burden and lower local IL-6 levels in GCF, whereas TNF blockade did not differ from control treatment. Although causality cannot be established due to the limited sample size, the findings are consistent with the hypothesis that IL-23–dependent immune pathways contribute to periodontal inflammation in IBD. Targeting this axis may therefore modulate inflammatory processes at both intestinal and oral mucosal surfaces. Future prospective, longitudinal studies with larger cohorts and detailed immunological profiling are warranted to validate these observations and to further elucidate the role of cytokine-targeted therapies in the oral–gut immune axis.

## Data Availability

The raw data supporting the conclusions of this article will be made available by the authors, without undue reservation.
